# The impact of interventions to prevent obesity or improve obesity related behaviours in children (0–5 years) from socioeconomically disadvantaged and/or indigenous families: a systematic review

**DOI:** 10.1186/1471-2458-14-779

**Published:** 2014-08-01

**Authors:** Rachel Laws, Karen J Campbell, Paige van der Pligt, Georgina Russell, Kylie Ball, John Lynch, David Crawford, Rachael Taylor, Deborah Askew, Elizabeth Denney-Wilson

**Affiliations:** Centre for Physical Activity and Nutrition Research, Deakin University, 221 Burwood Highway, Burwood, VIC 3125 Australia; Faculty of Health, University of Technology, Sydney, NSW Australia; School of Population Health, University of Adelaide, Adelaide, SA Australia; Inala Indigenous Health Service, Inala, QLD, Australia; University of Otago, Dunedin, New Zealand; Centre for Obesity Management and Prevention Research Excellence in Primary Health Care (COMPaRE-PHC), Kragujevac, Australia

**Keywords:** Obesity prevention, Children, Socioeconomically disadvantaged, Indigenous

## Abstract

**Background:**

Children from disadvantaged families including those from low socioeconomic backgrounds and Indigenous families have higher rates of obesity, making early intervention a priority. The aim of this study was to systematically review the literature to examine the effectiveness of interventions to prevent obesity or improve obesity related behaviours in children 0-5 years from socioeconomically disadvantaged or Indigenous families.

**Methods:**

Searches of major electronic databases identified articles published from 1993–2013 targeting feeding practices, anthropometric, diet, activity or sedentary behaviour outcomes. This was supplemented with snowballing from existing reviews and primary studies. Data extraction was undertaken by one author and cross checked by another. Quality assessments included both internal and external validity.

**Results:**

Thirty-two studies were identified, with only two (both low quality) in Indigenous groups. Fourteen studies had a primary aim to prevent obesity. Mean differences between intervention and control groups ranged from -0.29 kg/m^2^ to -0.54 kg/m^2^ for body mass index (BMI) and -2.9 to -25.6% for the prevalence of overweight/obesity. Interventions initiated in infancy (under two years) had a positive impact on obesity related behaviours (e.g. diet quality) but few measured the longer-term impact on healthy weight gain. Findings amongst pre-schoolers (3–5 years) were mixed, with the more successful interventions requiring high levels of parental engagement, use of behaviour change techniques, a focus on skill building and links to community resources. Less than 10% of studies were high quality. Future studies should focus on improving study quality, including follow-up of longer-term anthropometric outcomes, assessments of cost effectiveness, acceptability in target populations and potential for implementation in routine service delivery.

**Conclusion:**

There is an urgent need for further research on effective obesity prevention interventions for Indigenous children. The findings from the growing body of intervention research focusing on obesity prevention amongst young children from socioeconomically disadvantaged families suggest intervention effects are modest but promising. Further high quality studies with longer term follow up are required.

**Trial registration:**

PROSPERO Registration no: CRD42013006536.

## Background

Childhood overweight and obesity remains a significant public health challenge [1, 2]. In 2011–12, just over a quarter (25.1%) of Australian children aged 2–17 years were overweight or obese [3]. Increasingly, children are becoming overweight at a relatively young age which increases the risk of becoming an overweight adult, with the associated health consequences [4]. In 2011–2012, 17.8% of Australian children aged 2–4 years were overweight (BMI >=85 percentile on World Health Organisation BMI-for age growth charts) and 5.0% obese (BMI >=95 percentile on World Health Organisation BMI-for age growth charts) [3] Latest data from the USA for 2009–2010 indicates that in children aged two to five years, over a quarter (26.7%) were overweight (BMI > =85 percentile on Centre for Disease Control BMI-for-age growth charts) and 12.1% obese (BMI > =95 percentile on Centre for Disease Control BMI-for-age growth charts) [5]. Globally, the prevalence of overweight and obesity amongst preshoolers (aged 3–5 years) has increased dramatically since the 1990s, confirming the need for effective interventions commencing in early life [6].

In high-income countries, children from disadvantaged families including those from low socioeconomic backgrounds and Indigenous families have higher rates of obesity [7], making early intervention particularly important in these groups. Socioeconomic status is typically conceptualised as the social standing of an individual or group in society and is often measured by indicators such as education, occupation or income or a combination of these [8]. In a large representative sample of Australian children aged 4–5 years, Indigenous status and socioeconomic disadvantage were the clearest independent predictors of overweight and obesity [7]. Indigenous children were estimated to be 50% more likely to be overweight or obese compared to non Indigenous children. Similarly, the most disadvantaged children (highest quintile for an area level indicator of disadvantage) were almost 50% more likely to be overweight or obese compared to the most advantaged children. This corresponded to a difference in the prevalence of overweight or obesity of eight percent between the bottom and top quintiles of disadvantaged [7]. This highlights the importance of focusing obesity prevention efforts on children from low socioeconomic backgrounds and Indigenous children.

Evidence is also mounting that early intervention for both Indigenous and socioeconomically disadvantaged children is critical. A recent study of urban Australian Aboriginal infants, for example, has shown that 37% were overweight or obese at two years of age, and those experiencing rapid weight gain in the first year of life were significantly more likely to be overweight and obese compared to those not experiencing rapid weight gain [9]. Consistent with these data, is evidence that other Indigenous populations including Native American and Alaskan Natives also experience higher rates of obesity and excessive weight gain in the first two years of life [10, 11]. Similarly a longitudinal study of Australian children found that the socioeconomic differentials already present at four to five years of age not only remained but had more than doubled by age 10 to 11 years [12]. This is consistent with data from the UK [13] and Canada [14] that suggest that socioeconomic differentials emerge during the preschool years and widen with age.

The reasons children from socioeconomically disadvantaged and Indigenous families have higher rates of obesity are complex and multifactorial. Evidence suggests that predictors of child obesity in early life, such as unhealthy infant feeding practices, poorer diet, and sedentary behaviours are more prevalent in these families. For instance, a recent longitudinal study amongst socioeconomically disadvantaged families in the United States found that unhealthy infant feeding practices, including early introduction of solids (<4 months of age), feeding infants predominately formula for the first six months and putting infants to bed with a bottle, were the primary mechanism mediating the relationship between socioeconomic disadvantage and early childhood obesity [15]. Early dietary patterns at 6 and 15 months of age were also found to be associated with sociodemographic characteristics in a longitudinal study of children in the UK [16]. A socioeconomic gradient has also been reported in child diet [17, 18] and television viewing [17, 19] with evidence that maternal diet [18] and home television environment [19] are key mediators. While less is known about the mediators of the relationship between Indigenous status and obesity in children, predictors of child obesity, including lower breastfeeding rates [20], poorer diets and sedentary behaviours are more prevalent amongst Indigenous children. This suggests that children from socioeconomically disadvantaged and Indigenous families have a higher exposure to an obesity promoting environment and may benefit from interventions promoting healthy behaviours early in life. It is also likely that these groups will require obesity prevention interventions tailored to the specific barriers faced by these families.

In recent years, the body of literature on obesity prevention interventions in early life has increased considerably and this has been the subject of a number of systematic reviews [21–26]. This review however is unique in that it focuses specifically on obesity prevention interventions aiming to improve obesity related behaviours in children zero to five years from socioeconomically disadvantaged and Indigenous families. The review also included a broad range of study types including non controlled studies, recognising the emerging nature of literature in this area. We are unaware of any other review which has systematically reviewed this evidence base. This study will provide important new insights into the evidence for obesity prevention in early life amongst these high risk population groups, in particular the most promising intervention strategies and settings.

## Methods

This systematic review adhered to the Preferred Reporting Items for Systematic Reviews and Meta-Analysis (PRISMA) statement [27] and has been registered on PROSPERO, an international database of systematic reviews in health and social care (Registration no: CRD42013006536).

### Study selection criteria

The review included intervention studies published between 1993 and November 2013 in healthy young children aged zero to five years from socioeconomically disadvantaged or Indigenous families targeting: 1) prevention of unhealthy weight gain and/or 2) obesity related behaviours including child diet, physical activity levels, sedentary behaviours and parental feeding practices associated with obesity (e.g. breastfeeding and early introduction of solids). We defined socioeconomically disadvantaged families and their children as those described as low socioeconomic status, low income, low education (high school or below), or from low income areas. While there is no universally agreed definition of socioeconomic disadvantaged, this definition is in line with common ways this is measured [8]. Studies of both high and low socioeconomic status groups were included if the findings were stratified by one or more socioeconomic indicators (e.g. education/income). A well accepted definition of Indigenous populations was used (“the experience shared by a group of people who have inhabited a country for thousands of years, which often contrast to those of other groups residing in the country for a few hundred years”) [28] and the review included studies of Indigenous populations from any country and Maori people in New Zealand.

Studies had to report on one or more of the following primary outcomes: anthropometric measures, child/family diet, parental feeding practices (e.g. breastfeeding, time of introduction of solids, feeding style), physical activity or sedentary behaviours. These were chosen on the basis of being important predictors of overweight in young children [29]. The review excluded obesity treatment interventions that recruited only overweight or obese children, given that these interventions recruit a different target group, and are likely to vary in their approach compared with prevention-based interventions. Interventions exclusively targeting breastfeeding, or children with a specific illness or co-morbidities (e.g. diabetes) were also excluded. There were no limitations placed on the length of follow up, study design or study quality.

### Search strategy and study selection process

A comprehensive literature search was conducted by one researcher (RL) using MEDLINE, EMBASE, CINAHL, Psycinfo, Scopus, ATSIHealth (Aboriginal and Torres Strait Islander Health Bibliography), FAMILY-ATSIS (Australian Family & Society Abstracts Database – Aboriginal and Torres Strait Islander Subset), Indigenous collection and RURAL (rural and remote health database). An initial search was carried out in July 2013 and repeated in December 2013 and limited to peer reviewed studies published in English in the past 20 years (1993–2013) when the rapid increase in the prevalence of child obesity was first being reported [30]. The search strategy was devised using the Population, Intervention, Comparison, Outcome (PICO) framework [31] as outlined in Table 1, with key search terms provided in appendix one. Search terms were mapped to appropriate subject headings and searched as key words in title and abstract in each database. Citation searching of relevant systematic reviews and studies identified in the primary searches was also undertaken, including examining papers which had cited primary studies. Key experts in Indigenous health research were also contacted to identify potentially relevant studies. All articles were imported into an endnote library and duplicates removed.

**Table 1:** **Outline of the search strategy according to the PICO framework**

Population	Children zero to five years and families from socioeconomically disadvantaged or Indigenous backgrounds
Intervention	Lifestyle counseling, health education, health promotion, primary prevention, early intervention, diet or physical activity intervention, family therapy, parenting intervention
Comparison	control group (e.g. RCT), non equivalent control group (e.g. quasi-experimental design), baseline level (e.g. before and after studies)
Outcome	Anthropometric, diet, physical activity, sedentary behaviours, or parental feeding practices related to obesity (e.g. breastfeeding, timing of introduction of solids)

An initial screen of titles and abstracts was undertaken to identify eligible studies. This resulted in three categories of articles: 1) articles appearing to meet the selection criteria; 2) unsure articles (potentially eligible but further information required); 3) excluded articles. The full text of potentially eligible articles (1 and 2) were retrieved and assessed for eligibility. A 10% sample of titles and abstracts of excluded studies (n = 414), were cross checked by another researcher (Pv) to check on the reliability of the screening process, no additional articles were identified in this process.

### Data extraction

A data extraction template was developed that included study characteristics, recruitment, participant characteristics, intervention design and setting, outcome measures and results and study conclusions. All published papers and supplementary material related to the study (e.g. protocol papers, reference to websites, long term follow up studies) were referred to when extracting data. One researcher (RL) extracted the data and another researcher (Pv) cross-checked the accuracy of data extraction. Differences in data extraction and interpretation were resolved through discussion.

### Quality assessment

The internal validity of studies were assessed according to the McMaster University quality assessment tool [32]. This involved using a six component rating scale to assess selection bias, study design, confounders, blinding, data collection methods, withdrawals and drop-outs. A rating of low, moderate or high was allocated to each component based on specific criteria outlined in the tool. A global quality assessment is based on ratings of the six components with a low quality rating defined as two or more weak ratings; moderate as less than four strong and one weak rating, and high as four strong and no weak ratings.

An external validity assessment tool previously developed by RL [33] was used to assess the generalisability of the studies. External validity refers to the extent to which findings from a study or set of studies can be generalised to populations or settings beyond those in the original study [34]. The tool was based on the quality rating criteria proposed by Green and colleagues [34] and included five main dimensions: 1) reach and representativeness (individuals); 2) reach and representativeness (settings); 3) implementation and adaptation (of intervention); 4) outcomes for decision makers; 5) maintenance and institutionalisation. Institutionalisation refers to the potential for implementation of the intervention in routine service delivery [34]. Included studies were coded according to whether they met each element (yes, no or not applicable). Two authors (RL and Pv) independently assessed internal and external validity, and any discrepancies were resolved through discussion.

## Results

**Figure 1:**
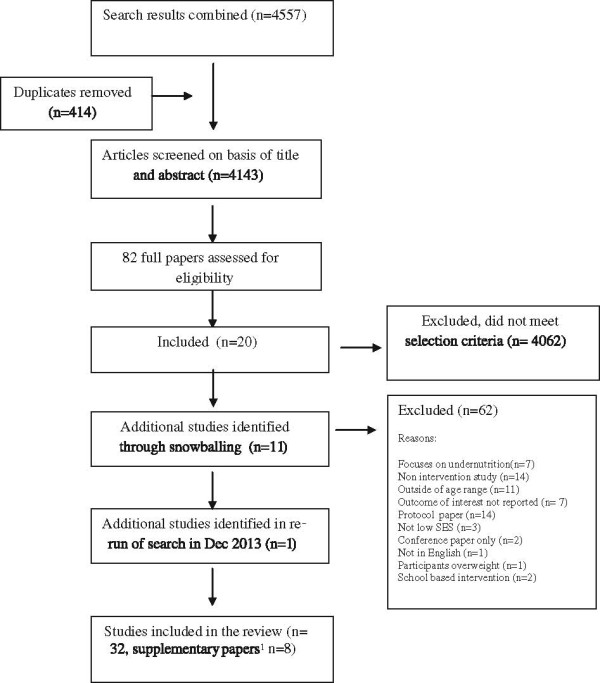
**Summary of search strategy and articles identified in the review.** ^1^Protocol papers or papers reporting long term follow up for primary studies included in the review.

A total of 4143 unique citations were identified through the search process and screened on the basis of title and abstract, and of these 82 full-text articles were assessed for eligibility. Thirty-one primary studies published between January 1993 and July 2013 met the eligibility criteria and one additional article was identified when the search was repeated in December 2013 (total studies n = 32). Twenty-two of these studies were identified through the searches of electronic databases and an additional 11 studies were identified through citation searching from the primary studies. An additional eight papers were identified that provided supplementary information (e.g. protocol papers or long term follow up of primary studies) (Figure 1).

### Study characteristics

Almost two thirds of studies (66%) used an RCT or cluster RCT study design, however the majority of studies were rated as moderate (44%) or low (47%) quality, with only three (9%) studies assessed to be high quality (internal validity assessment). Most studies were conducted in the USA (n = 22) or Europe (n = 7), among low income families. Just under half of all studies (n = 14) were conducted among racial minority groups, with Hispanic/Latino low income families being the most commonly studied group (n = 8). Of note, there were only two studies conducted amongst Indigenous populations (Native Indian and Native Alaskans). Interventions were most commonly conducted in the home (n = 12), primary health care (PHC) setting (n = 6), at preschool (n = 7) or in the community (n = 7). The delivery agents in half of all studies (n = 16) were either trained volunteers or paraprofessionals such as peer educators, doulas (non medical person who assists a woman before, during or after childbirth) and community health workers. One quarter of studies (n = 8) used health professionals to deliver the interventions (Table 2).

**Table 2:** **Study characteristics**

Study characteristics	Number (%) studies, n = 32
**Study design**	
RCT or cluster RCT	21 (65.6)
Quasi-experimental	6 (18.6)
Before and after	3 (9.4)
Prospective study with non equivalent comparison group	1 (3.1)
Simulated before and after design	1 (3.1)
**Quality**	
High	3 (9.4)
Moderate	14 (43.8)
Low	15 (46.9)
**Country where the study was conducted**	
USA	22 (68.8)
UK	5 (15.6)
Brazil	2 (6.2)
Switzerland	1 (3.1)
France	1 (3.1)
Australia	1 (3.1)
**Target population**	
Low income families (general population)	13 (40.6)
Hispanic low income families	8 (25.0)
Preschools with high migrant population	1 (3.1)
Low income racial minority families	1 (3.1)
Black preschool children and their families	2 (6.2)
Rural low income mothers	1 (3.1)
Adolescent low income mothers	1 (3.1)
Young (<22 years) Black low income mothers	2 (6.2)
Travelling (gypsy) mothers	1 (3.1)
Indigenous – Native Indian/Alaskan Native	1 (3.1)
Indigenous – Native Indian	1 (3.1)
**Delivery agents (n = 35)**	
Paraprofessionals (e.g. community health workers, health educators, peer educators, facilitators)	13 (40.6)
Trained volunteers	3 (9.4)
Dietitian/nutritionist	4 (12.5)
PHC staff (physicians, nurses and medical assistants)	2 (6.2)
Nurses	1 (3.1)
Multidisciplinary team	1 (3.1)
Preschool teachers	3 (9.4)
Trained child educators	2 (6.2)
Researcher	1 (3.1)
Play professional	1 (3.1)
Video only	1 (3.1)
Unclear	1 (3.1)

A summary of the studies included in the review can be found in Table 3 and the key findings presented below.

**Table 3:** **Summary of studies: focus, design, sample, delivery agent, quality assessment, and outcome**

Study/Target group/Aim	Focus/outcome measures							Design	N	Age at start	Delivery agent	Follow up	Quality	Outcome
	BF	PFP	Diet	PA	SB	Anthro	Theory based ^1^							
**Home Based (n = 12)**														
**Edwards et al**. (2013), low income young black mothers [36]	√	Delay solids						RCT	248	Antenatally	Doulas	Birth, 6 weeks, 4 m	Low	+BF, +solids
*1° Aim: Increase BF and delay early introduction of solids*														
**Black et al**. (2001), USA, low income black adoescents [46]		Delay solids					√	RCT	181	Birth	Trained mentors	3 &12 m	Moderate	+solids
*1° Aim: Delay early introduction of solids*														
**Wen et al**. (2012), Australia, first time mothers disadvantaged Community [37]	√	√	√	√	√	√		RCT	667	Antenatally	Nurses	6,12 & 24 m	High	+ BF, +PFP, +Diet, -PA, +SB, +Anthro
*1° Aim: Obesity prevention*														
**Scheinmann et al**. (2010), USA, low income Latino mothers [47]	√	Delay solids						Quasi-experimental	439	Infant < 5 months	Video only	3 & 6 m	Moderate	+solids
														-BF
*1° Aim: Delay early introduction of solids*														
**De Oliveira et al**. (2012), Brazil, low income adolescent mothers [38]	√	Delay solids	√					RCT	323	Birth	MD team	1,2,3,4,5 & 6 m	Moderate	+BF, +solids
														+ diet
*1° Aim: Delay early introduction of solids*														
**Vitola et al**. (2012), Brazil, low income mothers [39]	√	√	√			√		RCT	500	Birth	Trained field workers	6,12 m, 3–4 & 7–8 years	Moderate	+BF
														+diet
*1° Aim: Improve child diet*														-anthro
**Johnson et al**. (1993), UK, low income area [40]		√	√					RCT	262	<=4 months	Trained volunteers	12 m & 7 years	Moderate	+Diet
														+PFP
*1° Aim: Improved child development/diet*														
**Fitzpatrick et al**. (1997), UK, travelling mothers [35]		√	√					Prospective with non equivalent comparison	39	<=4 months	Trained volunteers	12 m	Low	+Diet
														+PFP
*1° Aim: Improved child development/diet*														
**Watt et al**. (2009), UK, low income mothers [41]	√	√	√			√	√	RCT	312	10 weeks age	Trained volunteers	12, 18 m & 4 years	High	-BF, -solids
														+diet, +PFP,
*1° Aim: Improve PFP and child diet*														-anthro
**Harvey-Berino** (2003), USA, Native American Toddlers/Pre-school children [49]		√	√	√	√	√		RCT	43	9 months to 3 years	Indigenous peer educators	4 m	Low	+PFP, -anthro, +diet, -PA
*1° Aim: Obesity prevention*														
**Karanja et al**. (2010), USA, American Indian/	√		√			√		Simulated before and after design	205	Antenatally	Community health workers	2 years	Low	?BF, diet NR,
														-anthro
Native Alaskan [48]														
*1° Aim: Obesity prevention*														
**Haines et al**. (2013), USA, low income racial minority families with preschool children [50]			√	√	√	√		RCT	121	2 to 5 years	Bi-lingual health educators	6 m	Moderate	+anthro
														+ sleep, +SB,
														-family meals, -TV in bedroom
*1° Aim: Obesity prevention*														
**Primary Health Care (n = 6)**														
**Kavanagh et al**. (2008), USA, low income WIC attendees [53]		√				√		RCT	61	3- 10 weeks	Unclear	4 m	Low	-formula feeding,
*1° Aim: Improve formula feeding practices and reduce rapid weight gain*														-anthro
**Kahn et al**. (2007), USA, predominantly low income Hispanic WIC attendees [54]		√					√	RCT	48	18-30 months	Nutritionist	2 m	Low	+bottle weaning
*1° Aim: Bottle weaning*														
**Klohe-Lehman et al**. (2007), USA, low income WIC attendees [57]			√	√		√	√	Before and after	235	1-3 years	Dietitian	2& 6 m	Low	+diet, +PA
														-anthro
*1° Aim: Improve diet and PA of mothers and children*														
**Davison et al**. (2011), USA, WIC attendees aged 2–5 years [56]				√	√			Quasi-experimental	880	2-5 years	WIC clinic staff	1 year	Low	+SB, +PA
*1° Aim: Increase child PA and reduce SB*														
**McGarvey et al**. (2004), WIC attendees 2–4 years [55]		√	√	√	√		√	Quasi-experimental	336	2-4 years	WIC nutritionist	1 year	Low	+PFP
*1° Aim: Improve child diet and PA, reduce SB*														
**French et al**. (2012), USA, low income families [51]		√	√		√	√		Cluster RCT	292	<2 months age	Primary care staff	12 m	Moderate	+ diet, -PFPs
														+ SB, -anthro
*1° Aim: Improve PFP and child diet*														
**Preschool (n = 7)**														
**Niederer et al**. (2012), Switzerland, Preschools with high migrant population [59]			√	√	√	√	√	Cluster RCT	652 (213 parents with low education)	5-6 years	Health educators and preschool teachers	1 year	High	-anthro, diet NR, PA NR, SB NR
*1° Aim: Improve aerobic fitness and obesity prevention*														
**Jouret et al**. (2009), France, Preschools [58]			√	√	√	√		Cluster RCT	79 kindergartens, 1663 children	2.5-5 years	Dietitian and education aide	2 years	Moderate	+Anthro
*1° Aim: Obesity prevention*														
**Fitzgibbon et al**. (2005), USA, Black preschool children [62]			√	√	√	√	√	Cluster RCT	12 preschools, 409 children	3-5 years	Trained child educators	2 years	Moderate	+Anthro -diet,
														-PA
*1° Aim: Obesity prevention*														
**Fitzgibbon et al**. (2006), USA, Latino preschool children [63]			√	√	√	√	√	Cluster RCT	12 preschools,	3-5 years	Trained child educators	2 years	Moderate	-anthro, -diet,
									401					-PA, -SB
*1° Aim: Obesity prevention*														
**Fitzgibbon et al**. (2011), USA, Black preschool children [64]			√	√	√	√	√	Cluster RCT	18 preschools, 618 children	3-5 years	Classroom teachers	14 weeks	Low	+PA, +SB, -diet, -anthro
*1° Aim: Obesity prevention*														
**Fitzgibbon et al**. (2013), USA Latino preschool children and their families [61]			√	√	√	√	√	Cluster RCT	4 preschools, 146 children	3-5 years	Bilingual educators	1 year	Low	-anthro, -diet,
														-PA, -SB
*1° Aim: Obesity prevention*														
**Winter et al**. (2013), USA, low income Latino preschool children [66]			√	√	√	√	√	Quasi-experimental	4 preschools, 405 children	3-5 years	Teachers, Promotoras	6 m	Low	-anthro, +PA
*1° Aim: Obesity prevention and school readiness*														
**Community setting (n=7)**														
**Horodynski et al**. (2004), USA, rural low income carers 1–3 year olds [68]			√					Quasi-experimental	38	1-3 years	Trained paraprofessional	6 m	Moderate	-diet
*1° Aim: Improve child diet*														
**Horodynski et al**. (2005), USA low income families with toddlers [69]		√	√					Quasi-experimental	135	11-25 months	Peer educator	6 m	Low	-PFP
*1° Aim: Improve child diet*														
**Davison et al**. (2013), USA, Head start attendees, 2-5 years [67]			√	√	√	√	√	Before and After	154	2-5 years	Trained facilitators	6 m	Low	+diet, +PA,
														+SB, +anthro
*1° Aim: Obesity prevention*														
**O’Dwyer et al**. (2012), UK, Sure Start attendees, 3–5 years [70]				√	√		√	Cluster RCT	77 families, 79 children	3-4.9 years	Researcher	10 weeks	Moderate	+PA
											Play professionals			+SB
*1° Aim: Improve child PA and reduce SB*														
**Barkin et al**. (2012), USA, Latino-American Preschool aged children [71]			√	√	√	√	√	RCT	106	2-6 years	Trained facilitator	3 m	Moderate	+anthro
*1° Aim: Obesity prevention*														
**Slusser et al**. (2012), USA low income Latino parents with 2–4 year olds [73]			√	√		√	√	RCT	160	2-4 years	Bilingual social worker	1 year	Moderate	+anthro
*1° Aim: Obesity Prevention*														
**Bender et al**. (2013), USA, low income Mexican parents with 3–5 year olds [72]			√	√		√	√	Before and after	33	3-5 years	Promotoras	15 m	Low	+diet, -anthro
*1° Aim: Improve child diet and maternal PA*														

BF: breastfeeding, PFP: Parental feeding practices, PA: physical activity, SB: sedentary behaviour, Anthro: anthropometrics, *1°*: Primary, ^1^theoretical basis of the intervention reported. Low quality: two or more weak ratings; moderate quality: less than four strong and one weak rating, high quality: four strong and no weak ratings, outcomes: +: significant effect, -: no significant effect, NR: not reported.

#### Home setting

There were a total of 12 studies delivered in the home setting (Table 3), four with the specific aim to prevent obesity, four focusing on parental feeding practices only and four targeting child diet and parental feeding practices. With two exceptions [35, 36], all of these studies were assessed as moderate to high quality. The majority of these studies (n = 7) were intensive home visiting interventions delivered antenatally [36, 37] from birth [38, 39] or early infancy [35, 40, 41] with 8–12 home visits in the first 12 to 24 months of the child’s life. Most of these studies (n = 5) used trained field workers and volunteers to deliver the intervention and only two studies used health professionals, with outcomes similar between delivery agents. Only studies commencing antenatally or at birth had a positive impact on breastfeeding outcomes, however all studies except one [41] (which recruited mothers when infants were around 10 weeks of age) were effective in delaying the introduction of solids. Similarly all studies had a positive impact on child diet at 12–24 months of age, with some [42–44] reporting longer term positive dietary outcomes. Just half of these studies [37, 43, 45] measured the impact of the intervention on child body mass index (BMI), with only one study by Wen and colleagues [37] reporting a statistically significant effect at 24 months. This equated to a mean BMI difference between intervention and control groups of -0.29 kg/m^2^ (95% CI: -0.55 to -0.02 kg/m^2^) and a difference in prevalence of overweight or obesity of 2.9% (95% CI: -3.0 to 8.3%). The other studies reported no impact on BMI with follow-up at four years [43], and seven to eight years [45].

Two studies [46, 47] specifically focused on delaying the introduction of solids to four to six months of age among ethnic minority groups (low income black adolescents [46] and low income Latino mothers [47]). These studies used tailored intervention strategies delivered by video only in one study and video plus home visiting in another. There was a statistically significant intervention effect in both studies. Black and colleagues [46] reported that low income black adolescent mothers receiving the intervention were nearly four times more likely (OR: 3.8, 95% CI: 1.6-9.1) to adhere to American Academy of Pediatric guidelines (introduce solids between four and 6 months of age), compared with mothers in the control group. Scheinmann and colleagues [47] found that the video group had a later age of introduction of solids than the control group (5.2 versus 4.9 months, P = 0.02).

Only two low quality studies were conducted among Indigenous populations with some promising findings that require confirmation in larger, better designed, randomised controlled trials. The toddler overweight and tooth decay prevention study (TOTS) [48] compared the feasibility and effectiveness of a community wide intervention, alone or in combination with intensive home visiting from birth among three distinct tribes of American Indian/Alaskan native families. The home visiting consisted of seven to 21 visits delivered by community health workers over first two years of the child’s life with half of all visits occurring in the first three months, with a strong focus on supporting breastfeeding. Community wide strategies include social marketing media campaigns and changes to public health practices (e.g. hospitals becoming more ‘baby friendly’, eliminating free formula packs) and policy changes (e.g. replacing sugar sweetened beverages with water at events where children were present). The impact on breastfeeding was mixed, with increased initiation and six month breastfeeding rates (compared to national average) in Tribe A (community alone) and Tribe B (community + home visiting) but not in Tribe C (community + home visiting). Compared to a pre-test sample of children of a similar age two years before the study began, BMI-Z scores increased in all tribes. However, the increase was less in Tribes B and C (community + home visiting) compared to Tribe A (community alone). Another small study [49] among Native American toddlers (n = 43, mean age 22 months) and their families used a 16-week home visiting program (1 hour per week) delivered by an Indigenous peer educator. The intervention focused on role modelling healthy behaviours, parental feeding practices and general parenting skills to set rules and routines around food, physical activity and TV watching. The intervention was effective in improving parental feeding practices (less use of restrictive feeding), reducing child energy intake and a weak trend of decreases in weight-for height z scores compared to general parenting support alone. The study was however underpowered and had only a short follow up duration of four months.

Only one home based study [50] (moderate quality) was conducted among preschoolers (mean age four years). This study focused on promotion of four household routines (family meals, adequate sleep, limiting TV time, no TV in the bedroom) among racial minority families (33% Black, 52% Hispanic). The intervention was delivered by bilingual health educators through four home visits and phone calls and one to two reinforcing text messages per week over six months. The intervention improved sleep duration, and decreased TV viewing and BMI at six months follow up (mean BMI difference between intervention and control groups of -0.40 kg/m^2^ 95% CI: -0.79-0.00 kg/m^2^).

#### Primary health care setting

Six studies were conducted within the PHC setting. Of these, five were implemented in The Special Supplemental Nutrition Program for Women, Infants, and Children (WIC), and only one study in general practice (pediatric primary care clinics). None of these studies had a primary aim to prevent obesity, but rather focused on specific parental feeding practices or improving child diet, physical activity levels or reducing sedentary behaviours. All primary care based studies were conducted in the USA, all, with the exception of one study [51] were assessed as low quality and none measured changes in BMI.

WIC agencies in the USA serve a high percentage of low-income families with children aged zero to five years and are organisationally positioned to deliver obesity prevention interventions as nutrition education and counselling at WIC clinics is a core component of program delivery [52]. All studies implemented in the WIC setting were delivered by WIC staff as part of routine service delivery. Two WIC studies specifically focused on aspects of bottle-feeding using brief interventions. Kavanagh and colleagues [53] reported that a brief educational intervention (one 45–60 minute session) had no impact on formula feeding practices and actually promoted weight gain compared to the control condition, despite improvement in mothers knowledge of infant satiety cues. The study was under-powered, had a short duration of follow up (four months) up and was a low intensity intervention. In contrast, a brief one-off counselling intervention by a WIC nutritionist using a weaning protocol was effective in reducing the total number of bottles consumed by toddlers (mean age 25 months) from predominantly Hispanic families [54].

The two WIC studies targeting pre-schoolers [55, 56] were effective in influencing obesity related behaviours or parenting practices. Davison and colleagues [56] reported that the provision of a community physical activity guide to parents as part of routine well child visits was effective in increasing child physical activity and reducing sedentary behaviours. Individual and group education sessions with WIC nutritionists was also effective [55] in increasing the frequency of active play and parents offering water to children. Both studies were limited by reliance on self reported data and use of quasi-experimental study designs that employed non randomised comparison groups.

Only one WIC study specifically targeted mothers as agents of change [57]. Klohe-Lehman and colleagues found that a weight loss program for overweight and obese mothers of one to three year olds was not only effective in promoting maternal weight loss and improving maternal diet and physical activity, but also had an effect in reducing the energy intake of their children and improving child diet quality and physical activity levels. Similarly, the one general practice based study [51] found that a mother focused intervention was as effective as a child focused intervention in improving child diet quality at 12 months of age compared to usual care. There was no effect of either intervention on infant weight gain and the study did not report the impact of either intervention on maternal outcomes.

#### Preschool setting

There were seven studies conducted in the preschool setting, all of which had a primary aim to prevent obesity. Two studies were conducted in Europe and five in the USA. The results of these studies were mixed and depended on the intervention mode, context and populations studied. Both of the European studies conducted post-hoc sub-group analysis to examine the effectiveness of their interventions for children from underprivileged areas [58] and parents of low education levels [59]. Jouret and colleagues [58] (moderate quality study) reported that weight screening, providing feedback to parents including basic information on overweight and health, and referral of overweight and obese children to their usual physician for management was effective in lowering BMI in preschool children from underprivileged areas (median change from baseline in BMI z score 0.35, 95% CI: -0.19 to 1.04 and 1.35, 95% CI: 0.57 to 1.82 for intervention and control groups respectively), Furthermore there was no change in prevalence of overweight/obesity in intervention group compared to a 22.6% increase in the control group. In contrast, children from more privileged areas benefited more from the addition of a preschool based educational component to the basic weight screening and referral strategy.

The Ballabeina trial in Switzerland [60] (high quality) found effects on diet, physical activity and body fat of a multi-component preschool based intervention that included a classroom based education/physical activity component, changes to the preschool environment and parental component consisting of three discussion sessions. Despite not being designed or powered for sub group analysis [59], the study revealed that children of parents with low levels of education (at least one parent with no education beyond mandatory schooling) benefited less from the intervention (average intervention effect size for BMI for children of parents with middle/high education level -0.11 kg/m^2^, 95% CI: -0.29 to 0.08 compared to 0.04 kg/m^2^, 95% CI: -0.15 to 0.23, for children of parents with low levels of education). Differences between high and low education groups did not reach statistical significance.

There were four studies reporting the outcomes of The Hip Hop to Health Jr intervention [61–64]. This program comprised a 40 minute educational intervention at preschool (20 minutes of physical activity and a 20 minute lesson focusing on nutrition messages) delivered three times a week for 14 weeks, supplemented with newsletters and homework assignments for parents who received a small monetary incentive for completing them [65]. The intervention had a positive impact on BMI amongst Black preschool children at one and two years follow up [62] when delivered by trained child educators (moderate quality, mean difference in BMI between intervention and control groups at two years of -0.54 kg/m^2^, 95% CI: -0.98 to -0.10). An effectiveness trial (low quality) using classroom teachers to deliver the intervention also showed a positive short term impact at 14 weeks on child physical activity and sedentary behaviour, but not on diet or BMI among Black preschool children [64]. Interestingly, when the intervention was delivered to Latino preschool children it was not effective and the authors posited that the parental component of the intervention may not have been intensive enough for the sample of low-acculturated Latinos [63]. In response to this, Fitzgibbon and colleagues pilot tested a ‘family-based Hip Hop to Health’ for Latino families [61] (low quality). This consisted of the standard Hip Hop intervention combined with a more intensive parental component consisting of six 90 minute group education and physical activity sessions for parents. However, attendance at the parental sessions was low (only 38% parents attended at least one session) and the intervention had no impact on child diet, physical activity, sedentary behaviours or BMI at one year compared to a general health intervention [61]. This suggests that parental engagement in preschool based interventions is critical to their success and that capacity to engage may differ by cultural group or be influenced by the cultural appropriateness of the program.

The “Healthy and Ready to Learn” study [66] (low quality) was also amongst Latino preschool children and used a unique intervention approach focusing on both obesity prevention and school readiness. The intensive six month intervention consisted of activities for children at preschool and at home as well as monthly training sessions for parents and 20 hours of training for preschool teachers. The intervention integrated nutrition and physical activity messages into activities to promote literacy (e.g. story telling) as well as focusing on physical activity sessions and gross motor development. The parental component focused on motivating parents to engage in health promoting behaviours and modelled how to implement child activities at home. The intervention had a positive impact on gross motor skills, physical activity and receptive language development (an important indicator of school readiness) but not BMI at six months follow up.

#### Community setting

Seven community-based studies were included in the review (three low and three moderate quality). Four of these studies [67–70] recruited and delivered interventions through federally funded programs promoting health and school readiness to low income families (Head Start in the USA and Sure Start in the UK) and three US studies [71–73] focused on community based interventions for Latino families with preschool age children.

Horodynski and colleagues published two studies [68, 69] reporting the findings of the Nutrition Education Aimed at Toddlers (NEAT) program delivered to low income families attending the Head Start program in the USA. The program consisted of group education sessions for parents focused on knowledge and skill acquisition. In the first study [68], six month follow up of the intervention comprising three 90 minute group education sessions revealed there was no difference between intervention and control groups in caregivers knowledge, attitudes or feeding practices or toddler diet. In a second study [69], the addition of an extra group session (four in total) and home visiting by a peer educator over six months had no impact on parental self efficacy or meal time behaviours despite demonstrated improvements in knowledge.

In contrast, two other community based studies amongst low income families with pre-schoolers showed positive impact of parent focused interventions. Davison and colleagues [67] used community-based participatory research to engage parents in developing and testing an intervention for Head Start families with children aged two to five years. The multi-component intervention included letters to parents reporting child BMI, a health communication campaign, informal nutrition counselling integrated into Head Start family events, six-weekly two-hour education program for parents and child recreational activities. The focus of this program was not only on improving awareness and knowledge, but on enhancing skills in social networking, media literacy, conflict resolution, communication skills and empowerment to access local resources. The pilot study showed that compared with pre intervention, children post intervention had significant improvements in diet quality, physical activity, TV viewing and rates of obesity decreased by 3.9%. O’Dwyer and colleagues [70] also reported that a 10 week family focused intervention with an activity and education component was effective in improving physical activity and reducing sedentary behaviour post intervention among preschoolers attending Sure Start program in North West England. This program included self-monitoring, use of behavioural contract and progressive reward systems and involved both parents and children.

Three community based studies [71–73] were conducted amongst Latino families with preschool aged children. These studies all used group education sessions for parents (ranging from four to 12 sessions over a two to three month period). Common features of the sessions were a focus on skill building (e.g., cooking, and parenting), behaviour change strategies (including goal setting and self monitoring), building social networks and accessing local community resources. These programs successfully engaged parents as evidenced by high rates of attendance at the group sessions. Two of the larger studies (n = 106 and n = 160) with moderate quality ratings had a positive impact on BMI at three months (mean BMI difference between intervention and control of -0.54 kg/m^2^) [71] and one year [73] (decrease of 9.1% in prevalence of overweight and obesity in the intervention group compared to a 16.3% increase in the control group), with the smaller low quality study demonstrating positive changes in diet following the intervention [72].

### Quality assessment

A summary of the quality assessment ratings across studies for each dimension of internal validity is provided in Table 4. Strengths of studies included in this review were strong study design with most (22/32) studies being randomised controlled trials and appropriate control for confounders. Key methodological weaknesses of studies were poor reporting of the validity and reliability of data collection tools used, with more than half of all studies (19/32) failing to report this, along with a high probability of selection bias in around half (15/32) of all studies. The selection bias related to both the methods for recruitment and in some studies the low proportion of eligible participants who agreed to take part.

**Table 4:** **Quality assessment ratings for included studies**

Quality component	Strong rating No (%)	Moderate rating No (%)	Weak rating No (%)
Selection bias	11/32 (34.3)	6/32 (18.8)	15/32 (46.9)
Study design	22/32 (68.8)	7/32 (21.9)	3/32 (9.4)
Confounders	21/32 (65.6)	0/32 (0.0)	11/32 (33.3)
Blinding	3/32 (9.4)	20/32 (62.5)	9 (28.1)
Data collection methods	13/32 (40.6)	0/32 (0.0)	19/32 (59.4)
Withdrawal and drop out	13/32 (40.6)	14/32 (43.8)	5 (15.6)
**Global rating**	**3/32 (9.4)**	**14/32 (43.8)**	**15/32 (46.9)**

Low quality: two or more weak ratings; moderate quality: less than four strong and one weak rating, high quality: four strong and no weak ratings.

### External validity

The external validity assessment of the studies is provided in Table 5. The target populations and settings were well described, however few studies (n = 3) reported on the representativeness of participants or how they recruited the sites within each setting (n = 2) and the representativeness of participating sites (n = 0). All studies described the intervention components and most detailed the delivery agent, the time required to deliver the intervention and the degree of intervention exposure. However, few studies reported how they recruited the delivery agents, their participation rates, how they were trained, or whether the fidelity of intervention delivery was measured. In terms of providing information for decision makers the reporting of outcomes compared to standards and attrition rates was high; however few studies reported information on cost or cost effectiveness, the dose response effect of the intervention or whether intervention effects were moderated by participant characteristics or delivery agents. The long term effects of the intervention, it’s ability to be implemented routinely (institutionalisation) and acceptability were reported in around half of all studies. No studies reported on all external validity dimensions.

**Table 5:** **External validity dimensions for included studies**

External validity dimension	No (%) studies
**Reach and representiveness of participants**	
Target population described	32 (100)
Methods to recruit target population described	23 (72)
Individual inclusion and exclusion reported	23 (72)
Enrolment rate	20 (63)
Representiveness of participants described	3 (9)
**Reach and representiveness of settings**	
Target setting described	32 (100)
Methods to recruit target setting described	2 (6)
Setting inclusion and exclusion reported	6 (9)
Setting participation rate	4 (13)
Representiveness of settings described	0 (0)
**Implementation and adaptation**	
Intervention characteristics described	32 (100)
Time to deliver intervention described	25 (78)
Intervention exposure reported	22 (69)
Delivery agent described	30 (94)
Method to recruit delivery agent described	1 (3)
Delivery agent participation rate described	0 (0)
Training of delivery agent described	11 (34)
Intervention fidelity measured	5 (16)
**Outcomes for decision makers**	
Outcomes compared to standards	26 (81)
Adverse consequences reported	5 (16)
Effect moderator by participant characteristics	5 (16)
Effect moderator by setting/delivery agent	0 (0)
Dose response effect of the intervention	1 (3)
Intervention costs or cost effectiveness	3 (9)
Attrition rates reported	29 (91)
Differential attrition rates reported	7 (22)
Representiveness of completers/drop outs	8 (25)
Long term effects (>6 months)	17 (53)
Acceptability	16 (50)
Institutionalisation	13 (41)

## Discussion

### Key findings

Obesity prevention interventions amongst young children from disadvantaged families is a rapidly growing area of research with almost all studies (29 out 32) being published in the past ten years and the majority (58%) being published in the past five years. However, less than 10% of the research reviewed here were of high quality. A high proportion of studies were conducted among racial and ethnic minority groups in the USA, however only two small low quality studies were identified among Indigenous populations.

Twenty of the 32 studies measured anthropometric outcomes, however only fourteen of these studies had a primary aim to prevent obesity. Of the six studies that recruited children before age two and measured anthropometric outcomes, only one study [37] (high quality) had a small effect on BMI (mean BMI difference -0.29 kg/m^2^ 95% CI: -0.55 to -0.02 kg/m^2^). This was equivalent to a 2.9% (95% CI -3.0 to 8.3%) difference between intervention and control groups in the prevalence of overweight and obesity at age two years. Given that there was an eight percent difference in the prevalence of overweight and obesity amongst Australian preschoolers between the top and bottom quintiles of disadvantage [7], this difference may be important in reducing the socioeocnomic ‘gap’ in obesity. The lack of impact of studies recruiting children before two years on anthropometric outcomes may be explained by a number of factors. These include obesity prevention was not the primary aim of four out of five of the negative studies, these studies largely focused on parental feeding practices and child diet, none focused on physical activity and only one of these studies [51] focused on sedentary behaviours. With two exceptions, [39, 41] these studies also had short term follow up which may not have allowed sufficient time to see the impact of the interventions on anthropometric outcomes.

Six of the 14 studies amongst two to five year olds that measured anthropometric outcomes reported statistically significant effects. Two of these studies [57, 72], did not have obesity as the primary outcome. The impact of interventions amongst two to five year olds was greater than those in children under two years, with the mean BMI difference between intervention and control groups ranging from -0.40 kg/m^2^ [50] to -0.54 kg/m^2^ [62, 71] in three studies of moderate quality. In another study [58] (moderate quality) mean difference in BMI z-score of 1, equating to a difference in prevalence of overweight and obesity of 22.6 percent between intervention and control groups.

Almost all of the studies (29 out of 32) reported a positive effect on at least one obesity- related behaviour such as child diet, physical activity, sedentary behaviour and/or parental feeding practices (e.g. breastfeeding or timing of introduction of solids). The few studies that reported no impact of the intervention on obesity related behaviours or practices focused largely on knowledge acquisition [68, 69] or had minimal parental component [63] or low levels of parental engagement [64].

### Recommendations for practitioners

The findings of this review provide important insights for practitioners about elements of effective interventions for socioeconomically disadvantaged parents, however the limited number of Indigenous studies prevent us making recommendations for interventions targeting this group. Anticipatory guidance approaches in infancy (generally from birth or antenatally) appear to be effective in influencing early obesity related behaviours such as breastfeeding or the timing of introduction of solids. Anticipatory guidance involves being proactive in providing support and advice to parents about what to expect and how to manage particular issues/situations before they occur [74, 75]. The findings of this review suggest however, that interventions need to commence in the antenatal period or at birth to positively impact on breastfeeding outcomes amongst socioeconomically disadvantaged mothers. Common features of successful interventions for pre-schoolers (aged three to five years) include a dual focus on obesity prevention and school readiness, weight screening and referral, focus on household routines and an educational component for parents. Studies with positive outcomes successfully engaged parents, had a strong focus on skill building (e.g. cooking skills, media literacy, communication, problem solving, conflict resolution and parenting skills), use of behaviour change strategies (such as self monitoring and goal setting), social networking, progressive rewards systems and links to community resources. Developing culturally appropriate programs appear to be critical to engaging parents from racial minority groups. Successful interventions also engaged children in educational activities related to nutrition, physical activity and sedentary behaviours as well as physical activity sessions focusing on development of gross motor skills.

This review also provides important insights into the type of setting in which to deliver interventions to socioeconomically disadvantaged families. Again, the limited number of Indigenous studies, prevent us making recommendations for settings to target Indigenous parents. The setting used for studies targeting socioeconomially disadvantaged parent in this review reflected the age of the child. For example, the home appears to be an effective setting to deliver interventions to infants under two years of age with all of these studies having positive effects on obesity related behaviours. PHC is also an emerging setting of interest for children under two years from socio economically disadvantaged parents, with three out of four studies conducted in children under two years showing positive outcomes, primarily delivered through the WIC program in the USA. Parents across all sociodemographic groups access PHC services frequently (on average twenty four visits in first year of life) [76] offering an unparalleled opportunity to reach the whole population and engage with disadvantaged families to support healthy parenting behaviours. Studies conducted in PHC settings in this review all used PHC providers to deliver the intervention as part of routine service delivery, increasing the chances of the intervention being sustained and delivered routinely. Federally funded programs promoting health and school readiness such as Head start in the USA, and Sure Start in the UK appear to be an effective setting for engaging with socioeconomically disadvantaged parents of preschool age children. The findings from preschool based interventions were mixed, with parental engagement being a critical factor to the success of interventions delivered in the preschool setting.

### Recommendations for future research

There are a number of recommendations for future research arising from this review. Firstly, given the paucity of studies focusing on Indigenous families, there is an urgent need for further research on effective obesity prevention interventions for Indigenous families around the world. This is particularly given the higher rates of overweight and obesity amongst young Indigenous children [9–11] and the associated increased risk of chronic disease in adulthood [77].

Secondly, the findings of this review point to the need for further studies targeting formula and bottle feeding practices. Only one study in this review targeted formula feeding practices of infants. Given the high rates of formula feeding amongst socioeconomically disadvantaged infants and that formula fed infants are significantly fatter than breastfeed infants at 12 months of age [78], further research is required to develop and test interventions targeting appropriate formula feeding practices. Similarly, only one study examined the effectiveness of a bottle weaning intervention in toddlers [54]. Given the association between prolonged bottle use and overweight [79, 80] and its high prevalence amongst low income groups [81], further research is required to confirm these findings using a larger more diverse sample.

Thirdly, an assessment of both internal and external validity of included studies point to a number of methodological recommendations for future studies. A key weakness in the internal validity of included studies related to poor reporting on the validity and reliability of measurement tools, particularly those related to measuring behavioural outcomes such as diet. It is recommended that objective measures are used wherever possible including the use of anthropometric outcomes. Future studies should also focus on reducing selection bias in the way participants are recruited. Inclusive recruitment methods that involve inviting all eligible participants in given target population or a random selection of participants is preferable to methods that rely on self referral or identification in a systematic way (e.g. participants attending a clinic). Researchers should also aim to report wherever possible, on the proportion of eligible participants agreeing to take part and on the representativeness of participants compared to non participants so the degree of selection bias can be assessed. Collection of data on non participants is challenging but may be possible using existing record systems (e.g. clinic) in some studies. The generalisability of studies in this review could also be improved by providing more information about delivery agents, particularly how they were recruited and trained, the costs of delivering the intervention, participant views on intervention acceptability and potential sustainability of the interventions. Given that most studies used intensive face-to-face interventions consisting of multiple sessions, information about acceptability, costs and potential for ‘institutionalisation’ of interventions is critical in advancing the transfer of research findings into routine practice. Future research should explore the feasibility, acceptability and effectiveness of low cost delivery modes such as used of mobile phone and internet based interventions. These modes of delivery have been found to be promising in promoting healthy lifestyle behaviours in children, adolescents [82] and adults [83] but remain under explored as an intervention delivery mode for child obesity prevention in young children.

### Review limitations

This review has a number of limitations. It only included peer-reviewed papers published in English over the past 20 years. There are unlikely however to be many studies published more than 20 years ago given that the majority of studies identified were published in the last five years. Publication bias may however have influenced the review findings in favour of positive outcomes. Studies from non English speaking countries may be under-represented and important findings published in grey literature may have been missed. The search strategy specifically focused on studies targeting socioeconomically disadvantaged or Indigenous children and may have missed studies that did not report on socioeconomic status. We did however, include studies of both high and low socioeconomic status groups were findings were stratified by one or more socioeconomic indicators (e.g. education/income). It was also not possible to combine the results of different studies in the form of a meta-analysis due to heterogeneity in the study populations, intervention types and outcome measures [84].

## Conclusion

There is a growing body of intervention research focusing on obesity prevention amongst young children from socioeconomically disadvantaged families. However less than 10% of the research reviewed here was of high quality. Future research should focus on using valid and reliable data collection tools and on reducing selection bias in the recruitment of participants. Moreover, there are only two low quality studies focused on how best to prevent unhealthy weight gain amongst Indigenous infants and young children. The findings of obesity prevention interventions amongst socioeconomically disadvantaged families are promising, when commenced in early infancy, although longer term follow up is required to assess the impact on healthy weight gain. Interventions amongst pre-schoolers including racial and ethnic minority groups are more effective when they have a strong component of parental engagement, use evidence based behavour change techniques, focus on building skills not just knowledge acquisition, provide rewards and links to social networking opportunities and community resources. Overall effect size of moderate to high quality studies was modest, with changes in BMI ranging from -0.29 kg/m^2^ to -0.54 kg/m^2^. Future research should be of higher quality in its design and focus on low cost delivery modes that have potential for implementation in routine practice so effective interventions have the potential for population level impact.
